# Keratoprosthesis: A Review of Recent Advances in the Field

**DOI:** 10.3390/jfb7020013

**Published:** 2016-05-19

**Authors:** Borja Salvador-Culla, Paraskevi E. Kolovou

**Affiliations:** Department of Ophthalmology, Massachusetts Eye and Ear Infirmary, Harvard Medical School; 243 Charles Street, Boston 02114, MA, USA

**Keywords:** keratoprosthesis, Boston keratoprosthesis, osteo-odonto-keratoprosthesis, osteo-keratoprosthesis, keraklear, miro cornea

## Abstract

Since its discovery in the years of the French Revolution, the field of keratoprostheses has evolved significantly. However, the path towards its present state has not always been an easy one. Initially discarded for its devastating complications, the introduction of new materials and the discovery of antibiotics in the last century gave new life to the field. Since then, the use of keratoprostheses for severe ocular surface disorders and corneal opacities has increased significantly, to the point that it has become a standard procedure for corneal specialists worldwide. Although the rate of complications has significantly been reduced, these can impede the long-term success, since some of them can be visually devastating. In an attempt to overcome these complications, researchers in the field have been recently working on improving the design of the currently available devices, by introducing the use of new materials that are more biocompatible with the eye. Here we present an update on the most recent research in the field.

## 1. Introduction

The basic concept of using an artificial cornea or keratoprosthesis to replace a damaged and opaque cornea is as obvious as placing a window on a house to be able to see out. This possibility first occurred to the French doctor Guillaume Pellier de Quengsy, who published the feat in the times of the French Revolution (18th century) [1,2,3]. However, at a time of no anesthesia, asepsis, or antibiotics, the outcomes of his early attempts were obviously disastrous, and the idea was abandoned for decades. During the 19th century there were scattered surgeons who attempted to follow on Quengsy’s footsteps, but with equally disastrous outcomes (endophthalmitis, extrusion, and loss of the eye). Thus, it was not until the 1950’s with the introduction of new materials, such as transparent non-toxic plastics, that some measure of success began to be reported [4,5,6,7]. The good results of these new designs has to be also attributed to the discovery of antibiotics and steroids, which improved the postoperative management significantly. Since then, the advancements in the field have continued to grow, mainly thanks to the development of new devices and, more importantly, the improvement of the surgical techniques in preserving the health of the surrounding corneal tissue. There have been many different models throughout the years that have been implanted and trialed in animals and humans [8]. The purpose of this review is not to describe all of them but to present the latest developments in the field.

Presently, there are three keratoprosthesis with a widespread use in humans: the Boston keratoprosthesis (B-KPro) [9,10], the osteo-odonto-keratoprosthesis (OOKP) [11,12,13], and the so-called MICOF (Moscow eye microsurgery complex in Russia) [14,15]. Likewise, newer designs are recently strongly emerging, such as the KeraKlear^®^ (Keramed) [16,17], the Miro Cornea^®^ [17,18], and the Alphacor [19,20]. However, while the first two have presented promising initial results despite a very short follow up, the latter has been recently discontinued after developing severe mid- and long-term complications. Therefore, the OOKP and the B-KPro still remain the reference models, the latter with over 12,000 units implanted worldwide (March 2015).

## 2. Recent Research on Boston Keratoprosthesis (B-KPro)

**Figure 1:**
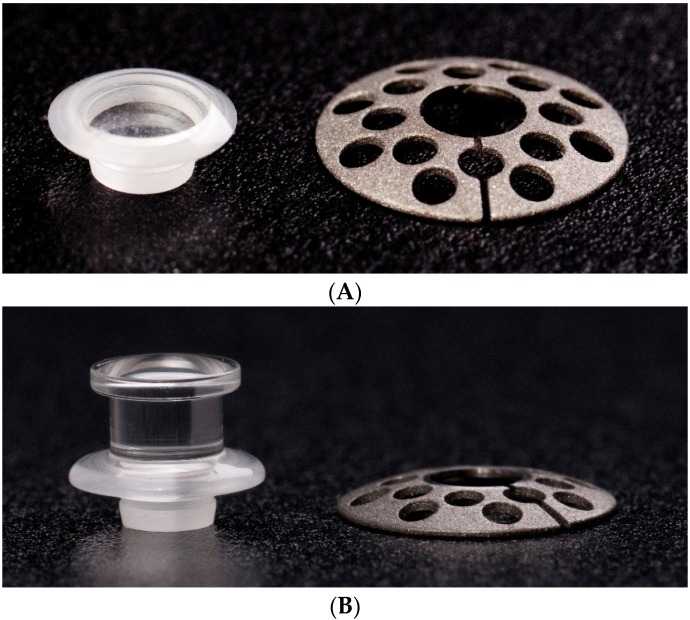
Boston keratoprosthesis (**A**) type I and (**B**) type II. Both models consist of a front plate with an optical cylinder of poly (methyl methacrylate) (PMMA) and a back plate of titanium. (Courtesy of Dr. Chodosh, Massachusetts Eye and Ear Infirmary).

Developed at the Massachusetts Eye and Ear Infirmary in the 1960s, approved by the FDA in 1992, and CE-marked in 2014, the Boston KPro type I is made of pure PMMA and designed to be implanted into a carrier corneal graft, like a collar button [10]. The device consists of two main parts: an anterior plate of poly(methyl methacrylate) (PMMA) (5.5 mm diameter) with a central optical stem of 3.35 mm in diameter, and a snap-on titanium back plate (7.0–8.5 mm diameter) with 16 holes (1.3 mm diameter each) that facilitate the access of the corneal tissue to the aqueous humor. A donor cornea is sandwiched between the plates, and the complex is then sutured into the patient’s own eye like a standard graft (Figure 1A) [21]. For cases with severe ocular surface disease, end-stage dryness, or incomplete blink, a newer design (type II) exists, with an anterior nub extension that emerges between the lids or through the upper lid (Figure 1B) [22].

**Figure 2:**
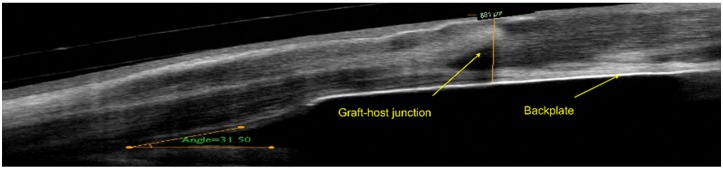
Anterior segment ocular coherence tomography (AS-OCT) image of a patient with a back plate of a relative bigger size than the donor cornea. The graft-host junction is perfectly aligned. (Courtesy of Dr. Cruzat, Massachusetts Eye and Ear Infirmary).

In the last decade, significant advances to the design of the B-KPro, as well as to the management of postoperative complications, have been implemented. The most important changes have been the introduction of a daily dose of a topical broad-spectrum antibiotic prophylaxis to reduce the rate of infectious keratitis and endophthalmitis [23], the introduction of holes on the back plate to allow better nutrition of the donor cornea from the aqueous humor and consequently reduce the rate of tissue melting [21], and the recent substitution of the PMMA by titanium as the main component of the back plate [24]. Other modifications have been focused in decreasing the rate of retroprosthetic membranes by using an enlarged back plate to clamp the donor-host junction (Figure 2) [25].

**Figure 3:**
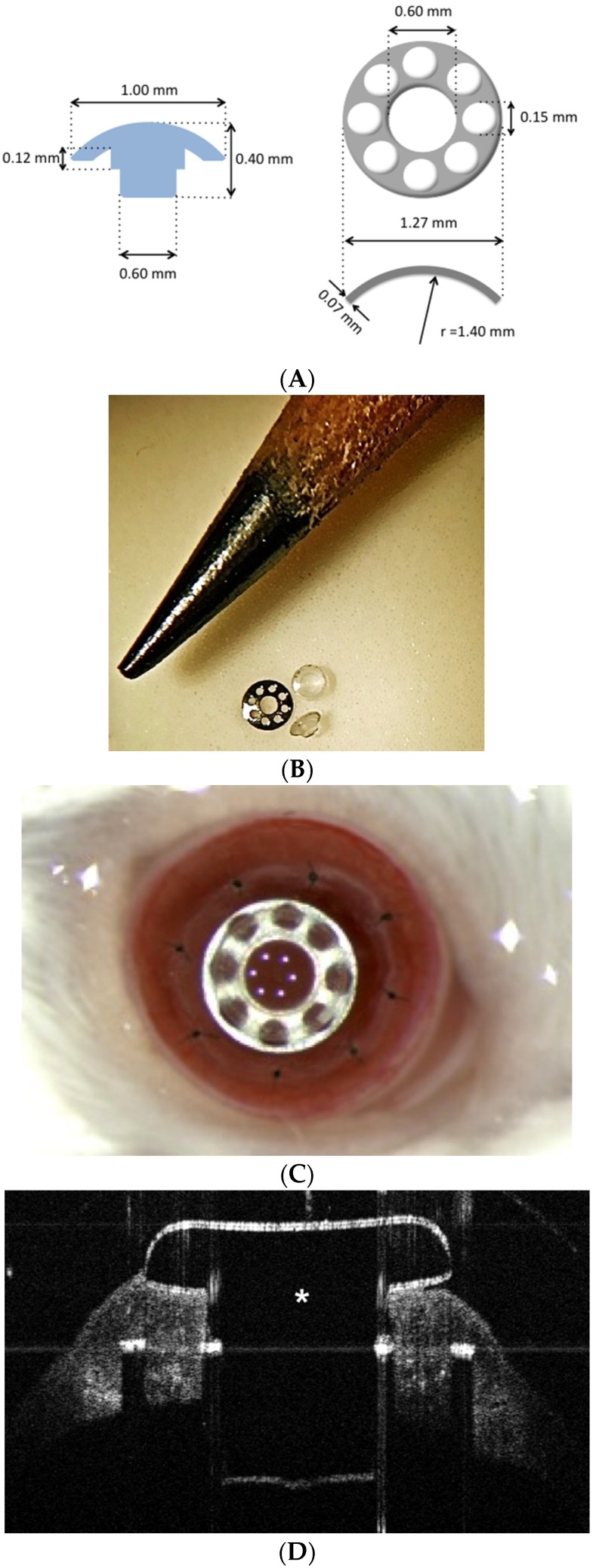
(**A**) Schematics of the miniaturized B-KPro (m-KPro); (**B**) real size image of a m-KPro; (**C**) image of a m-KPro one day after implantation in a mouse eye; and (**D**) AS-OCT of a m-KPro. The asterisk (*) indicates the optical cylinder. (Courtesy of Dr. Crnej, Massachusetts Eye and Ear Infirmary).

Currently, the post-operative complications after B-KPro can be grouped in four categories: infection, tissue necrosis/melt, glaucoma, and chronic inflammation. As mentioned above, the rate of infection and endophthalmitis has decreased significantly in the last decade since the introduction of a daily dose of antibiotic prophylaxis [23]. Likewise, the incidence of complications derived from a low-grade chronic inflammation, such as tissue necrosis and melt, epiretinal membrane formation or retinal detachment, have been also reduced significantly. However, there is still the need for a close follow up of these patients, especially since the clinical signs of chronic inflammation can be subtle and difficult to detect. In an attempt to address these issues, our group has recently developed a miniaturized model of the B-KPro (m-KPro) (Figure 3) that can be implanted in rodents. This new design has allowed us to study the levels of pro-inflammatory markers, such as cytokines and collagenases, after m-KPro implantation, and compare them to those after penetrating keratoplasty [26,27]. Along the same lines, we have explored the use of tumor necrosis factor-alpha (TNF-α) inhibitors, such as Infliximab^®^, in decreasing the levels of inflammation in patients with B-KPro after severe chemical burns [28].

Another serious complication after B-KPro is glaucoma. The presence of damage to the optic nerve can be oftentimes only confirmed after the device is implanted and the view to the back of the eye is restored. In a recent publication, our group has shown that 66% of patients presented glaucomatous changes prior to B-KPro implantation, while 24% did not show any signs of glaucoma. However, 75% of the latter still developed glaucoma *de novo* after B-KPro implantation. Moreover, the progression rate of optic disc excavation was similar in all cases, with the only exception of those patients who had a glaucoma shunt implanted, in whom the rate of progression was significantly decreased [29]. These revealing results have encouraged our group to try to find new therapeutic strategies through three different lines of investigation.

**Figure 4:**
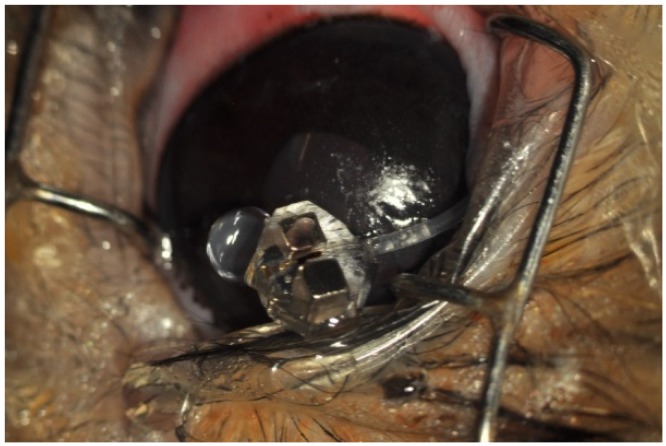
Image of the new prototype of glaucoma shunt implanted in a rabbit’s eye. The device is designed to be implanted in the lower fornix; for the picture, the valve has been displaced outside the fornix. Note the drop of aqueous humor, which confirms that the valve is open (opening pressure is 10 mmHg).

The first line has focused on the development of a new glaucoma shunt. Using ferrofluids and magnetism, our prototype has shown not only a clinically relevant opening pressure, but also a true closing pressure (Figure 4) [30]. The two values-opening and closing pressures-can be customized for each patient, based on the preferred targeted intraocular pressure (IOP), during the manufacturing process. The initial results in animals are promising, and we are currently in the process of optimizing the prototype before starting with the clinical trials in humans.

**Figure 5:**
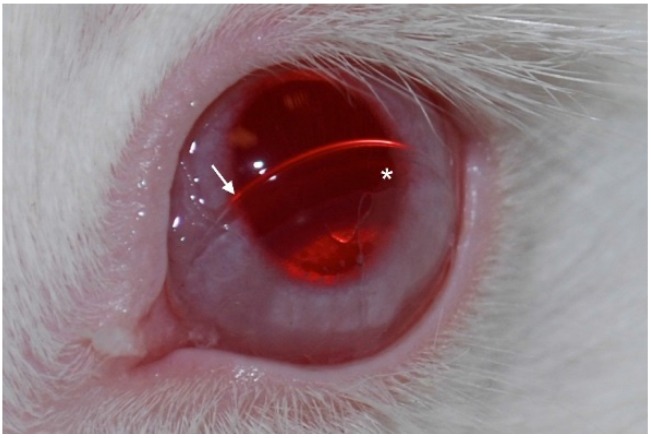
Image of one of our Latanoprost contact lens drug delivery device in a rabbit’s eye. The lens has been displaced slightly for a better visualization of the drug film. The arrow shows the edge of the contact lens; the asterisk indicates the drug film area. (Courtesy of Dr. Ciolino, Massachusetts Eye and Ear Infirmary).

The second line of investigation has focused on glaucoma medication and compliance. Thus, we have developed a new contact lens drug delivery device that can release sustained levels of Latanoprost in the anterior chamber for a minimum of 28 days, with a good tolerance and retention of the lens *in vivo* (Figure 5) [31]. We have recently finalized with the optimization studies in animals (manuscript under review), and will soon start with the clinical trials in humans.

The last line of investigation has focused on addressing the limitations of measuring the IOP in B-KPro patients. Due to the rigidity of the front plate, aplanation tonometry is not a viable option. For years, surgeons have relied on finger palpation, a very crude method, to assess IOP in these patients. Therefore, the development of a reliable method to measure IOP was greatly needed. Thus, a new experimental wireless intraocular pressure transducer (WIT), developed in Germany, that can be inserted into the cilliary sulcus following extracapsular lens extraction and “in the bag” intraocular lens insertion, has been recently successfully implanted in humans [32,33]. These initial results are promising, since the IOP could be reliably and repeatedly measured for at least 18 months after implantation, which allowed starting anti-glaucoma treatment as soon as a pathological increase of the IOP was detected [32].

**Figure 6:**
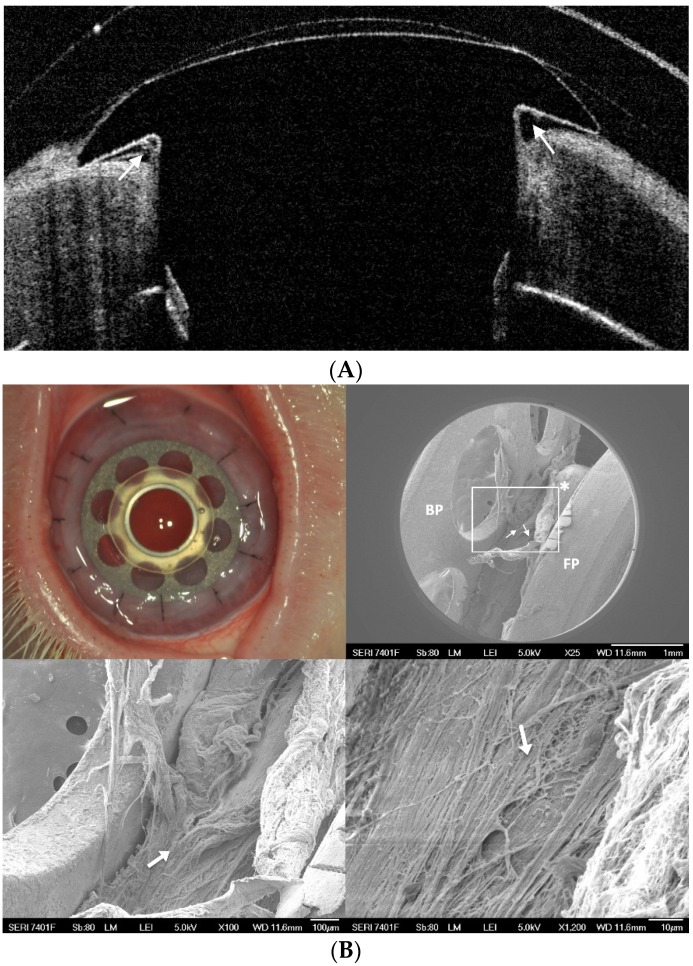
(**A**) Anterior segment optical coherence tomography of an eye with a B-KPro. Note the space between the stem and the cornea indicated by the arrows. (Courtesy of Dr. Cruzat, Massachusetts Eye and Ear Infirmary); and (**B**) sequence of images showing a B-KPro with a titanium ring around the stem after implanting it in a rabbit eye (upper left). The scanning electron microscopy (SEM) images taken ten months after implantation show the entire surface of the stem covered in corneal tissue, suggesting a strong adherence with the titanium ring. This can be clearly seen when the area enclosed by the rectangle and denoted by the * in the upper right image is magnified at 100× and 1200×, respectively, as indicated by the arrows in the bottom two images. BP: back plate. FP: front plate. Stem with a titanium ring (*). The arrows indicate the remnants of the corneal tissue adhered to the stem after removing the cornea.

The last group of complications after B-KPro implantation includes those related to tissue necrosis and melt. These events usually start around the stem of the B-KPro, and progress creating a space between the cornea and the device that in turn can facilitate microorganisms and debris to enter in the eye, and ultimately induce endophthalmitis. In an attempt to address this issue, our group has been investigating the use of new materials, such as hydroxyapatite [34] and titanium [35], to induce a stronger attachment between the cornea and the device. In a recent study, the authors have shown that by introducing a ring of sandblasted titanium around the stem, the adherence between the cornea and the B-KPro was significantly enhanced (Figure 6) [35]. We believe that by incorporating this approach, the likelihood of postoperative complications, such as keratolysis and endophthalmitis, can be significantly reduced.

**Figure 7:**
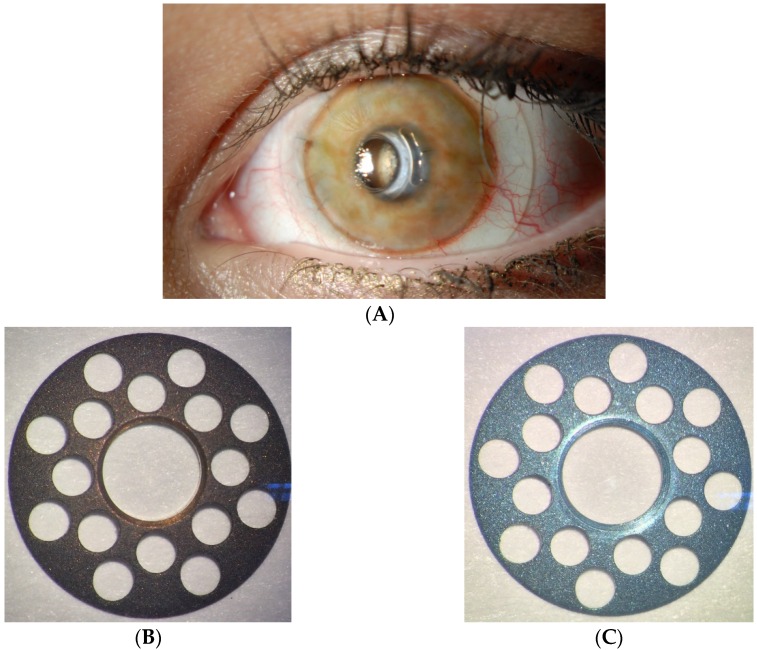
(**A**) Image of a B-KPro patient with a tinted contact lens in place; (**B**) brown-colored titanium back plate; and (**C**) blue-colored titanium back plate. (Courtesy of Dr. Dohlman and E. Paschalis, Massachusetts Eye and Ear Infirmary).

Finally, the cosmesis of the titanium backplate of the B-KPro, with a metallic blue shining color, has become a concern for some of our patients, especially those with brown or dark eyes. For years, we have recommended the use of a tinted contact lens (Figure 7A) [36]. Although effective, these lenses have their limitations and can become costly. In this way, we have been exploring the possibility of coloring the titanium back plate, without the need of dyes or other toxic chemicals, through a technique called ionic anodization [37]. In short, this technique adds an inert and biocompatible titanium oxide layer on the surface of the plate. The electric current applied during the process will determine the thickness of the oxide layer, which in turn will define the final color. Thus, we have explored the possibility of coloring the back plate brown (Figure 7B) or blue (Figure 7C), although other colors can be easily applied [37]. We believe that this approach will improve the cosmetics of the prosthesis by providing a back plate of a color that is close to the patient’s contralateral eye.

## 3. Recent Developments on Osteo-Odonto-Keratoprosthesis (OOKP)

**Figure 8:**
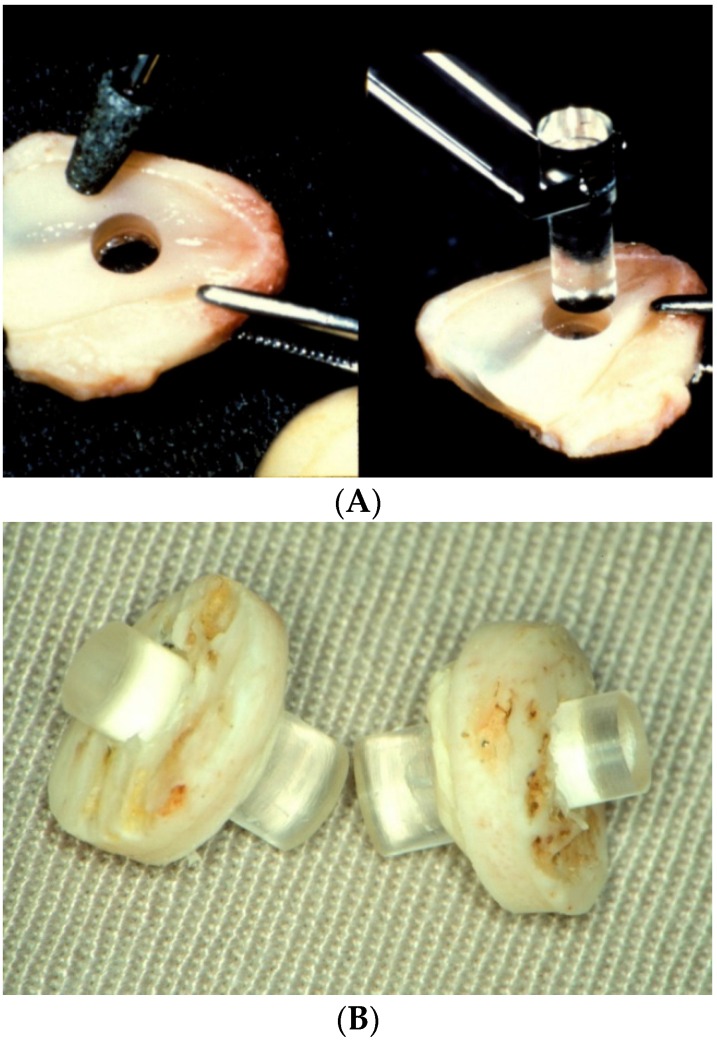
(**A**) Insertion of the optical cylinder through the tooth during the first step of an osteo-odonto-keratoprosthesis (OOKP) implantation. Note the difference in diameter between the anterior and the posterior segments; and (**B**) image of two assembled osteo-keratoprosthesis (OKP) prior to surgical implantation. (Courtesy of Dr. Temprano, Centro de Oftalmología Barraquer).

Strampelli introduced for the first time in 1963 the so-called osteo-odonto-keratoprosthesis (OOKP). The idea of using a tooth as a haptic for his new device was initially taken with skepticism by his contemporary colleagues [11]. Yet, the persistence of Strampelli, helped by the excellent initial visual results, turned OOKP in the keratoprosthesis of reference for decades. The prosthesis consists of an optical PMMA cylinder with two distinct segments: one anterior with a mean diameter of 3.65 mm (3.3–4.0 mm), and one posterior with a mean diameter of 4.1 mm (3.6–4.6 mm) [38]. The difference in diameter allows anchoring the optical cylinder to the tooth, in a way that the posterior segment acts as a stop, preventing the spontaneous extrusion of the cylinder (Figure 8A). The surgical procedure is performed in two or three steps separated by a period of 3–5 months each, and has been presented elsewhere [38].

Although the rate of complications initially reported by Stampelli was lower than that of his contemporaries, the OOKP was not without problems [38]. In an attempt to reduce these and improve the clinical results, Falcinelli introduced significant modifications to the initial design, including the use of a relative’s tooth, cryoextraction of the lens, anterior vitrectomy, the use of buccal instead of labial mucosa, and iris removal, among others [13]. Several years later, Temprano introduced another variation when he used a fragment of the tibia in a patient who lacked teeth. The so-called osteo-keratoprosthesis (OKP) (Figure 8B) presented comparable anatomical and visual outcomes, although reabsorption of the bone occurred more frequently, resulting in an increased rate of extrusion of the device [39].

In the last decade, research on OOKP has focused on finding new materials that could replace the tooth, while at the same time enhance the adherence between the optical cylinder and the surrounding tissues in the eye [40,41,42,43]. Likewise, the introduction of improvements on the surgical technique, such as using Mytomicyn C to prevent epithelial growth over the prosthesis [44], may reduce the rate of postoperative complications significantly and improve the long-term results.

## 4. What Next?

The advances shown herein are only a small sample of the boiling point in which the field of keratoprotheses has recently turned into. Current research is aimed at improving, on the one hand, the anatomical results by using more biocompatible materials that provide better integration with the host tissue, and on the other hand, at providing optimal long-term and sustained visual acuity to our patients. However, post-operative complications remain the great enemy to beat (mainly glaucoma, infection, and extrusion). Future designs will have to incorporate newer materials that provide excellent optical properties, while at the same time become biointegrated with the ocular tissue. In short, the perfect keratoprothesis has yet to be discovered, although every day the goal gets closer and closer.
